# New evidence that a large proportion of human blood plasma cell-free DNA is localized in exosomes

**DOI:** 10.1371/journal.pone.0183915

**Published:** 2017-08-29

**Authors:** M. Rohan Fernando, Chao Jiang, Gary D. Krzyzanowski, Wayne L. Ryan

**Affiliations:** 1 Department of Obstetrics and Gynecology, University of Nebraska Medical Center, Omaha, NE, United States of America; 2 Department of Research and Development, CFGenome®, Omaha, NE, United States of America; Universita degli Studi di Torino, ITALY

## Abstract

Cell-free DNA (cfDNA) in blood is used as a source of genetic material for noninvasive prenatal and cancer diagnostic assays in clinical practice. Recently we have started a project for new biomarker discovery with a view to developing new noninvasive diagnostic assays. While reviewing literature, it was found that exosomes may be a rich source of biomarkers, because exosomes play an important role in human health and disease. While characterizing exosomes found in human blood plasma, we observed the presence of cfDNA in plasma exosomes. Plasma was obtained from blood drawn into K_3_EDTA tubes. Exosomes were isolated from cell-free plasma using a commercially available kit. Sizing and enumeration of exosomes were done using electron microscopy and NanoSight particle counter. NanoSight and confocal microscopy was used to demonstrate the association between dsDNA and exosomes. DNA extracted from plasma and exosomes was measured by a fluorometric method and a droplet digital PCR (ddPCR) method. Size of extracellular vesicles isolated from plasma was heterogeneous and showed a mean value of 92.6 nm and a mode 39.7 nm. A large proportion of extracellular vesicles isolated from plasma were identified as exosomes using a fluorescence probe specific for exosomes and three protein markers, Hsp70, CD9 and CD63, that are commonly used to identify exosome fraction. Fluorescence dye that stain dsDNA showed the association between exosomes and dsDNA. Plasma cfDNA concentration analysis showed more than 93% of amplifiable cfDNA in plasma is located in plasma exosomes. Storage of a blood sample showed significant increases in exosome count and exosome DNA concentration. This study provide evidence that a large proportion of plasma cfDNA is localized in exosomes. Exosome release from cells is a metabolic energy dependent process, thus suggesting active release of cfDNA from cells as a source of cfDNA in plasma.

## Introduction

Extracellular vesicles released by cells are classified by their intracellular origin. The three main categories of vesicles are apoptotic bodies, microvesicles and exosomes. Apoptotic bodies are released from cells which undergo apoptosis. They contain intracellular fragments, cellular organelles and fragmented DNA [1, 2]. Apoptotic bodies are 1–5 μm in diameter, the same size range as platelets. Microvesicles are generated by outward budding of the plasma membrane. The size of microvesicles range is between 100–1000 nm in diameter. The intracellular origin of exosomes is endosomes. Exosomes are the best characterized extracellular vesicles. Their size range is between 30–100 nm in diameter. The content of exosomes is controlled by the cell thus reflecting the cellular origin and physiological state of the cell [3]. Exosome specific markers such as tetraspanins (CD81, CD63 and CD9), alix, TSG101 and flottilin can be used to differentiate exosoms from other extracellular vesicles. Exosomes have the ability to modulate immune system [4], transfer genetic material [5] and influence cells in long distance. Exosomes may carry out these functions through biomolecules present in exosomes.

Extensive studies have been conducted on the content of exosomes released by different cell types during the past ten years. Exosomes contain proteins that help cell penetration, invasion and fusion, proteins such as CD81, CD63 and CD9. Proteins such as Alix, TSG101 and clathrin found in exosomes are involved in exosome biogenesis [6]. In addition to proteins, exosomes also contain DNA, mRNAs and microRNAs. Exo-Carta (http://www.exocarta.org/) is an online exosome protein, lipid and RNA database. In this database there are 9,769 exosome proteins, 3,408 mRNAs, 2,838 miRNAs and 1,116 lipids. There is mounting evidence that exosomes are a rich source of biomarkers for new diagnostic test development. Recently, we have started a research project to characterize human blood plasma exosomes, with a view to develop new noninvasive diagnostic tests. In this exosome characterization study, we found evidence that more than 90% of cfDNA in human blood plasma is localized in plasma exosomes.

## Materials and methods

### Human blood samples

Blood samples were obtained from the American Red Cross Apheresis Center located at the University of Nebraska Medical Center from consented and certified as healthy, donors. Blood was drawn into 10 mL K_3_EDTA tubes (BD Vacutainer®, Becton Dickinson, Franklin Lakes, NJ) and all draws were performed using venipuncture.

### Plasma separation

For the separation of plasma, blood samples were centrifuged at 22°C at 1600 x g for 10 minutes. The plasma layer was carefully removed without disturbing the buffy coat, transferred to a new tube and then centrifuged at 22°C at 16000 x g for 10 minutes to remove residual cells, cell debris, apoptotic bodies, and nucleuses.

### Isolation of exosomes from cell-free plasma

Exosomes were isolated from cell-free plasma using two different methods. In one method Invitrogen Total Exosome isolation (from plasma) kit was used to isolate exosomes following manufacturer’s instructions. Briefly, 0.5 mL of plasma was placed in an Eppendorf tube and mixed with 0.25 mL of PBS. Diluted plasma was then treated with Proteinase K (25 μL) and incubated at 37°C for 10 minutes. Next, each plasma sample was combined with 150 μL of Invitrogen Total exosome isolation (from plasma) reagent and then mixed well by vortexing until a homogenous solution was formed. The samples were incubated at 4°C for 30 min and then centrifuged at room temperature at 10,000 × g for 5 minutes. The supernatant was aspirated and transferred to a new clean tube and stored at -20°C until use. The exosome pellet was re-suspended in PBS buffer and then stored at 4°C short term (1–7 days) or −20°C for long term. The second method used to isolate exosomes was a density gradient centrifugation as previously described by Kalra et al. [7]. First, exosomes were isolated from diluted plasma using a combination of differential centrifugation and ultra-centrifugation as desceribled. The exosome pellet obtained was further purified using density gradient centrifugation. Briefly, a discontinuous OptiPrep™ (60% w/v aqueous iodixanol from Sigma Life Sciences®) gradient consisting of 40% w/v, 20% w/v, 10% w/v, and 5% w/v solutions were prepared in 0.25 M sucrose/10 mM Tris, pH 7.5. The gradient was prepared by layering of 3 mL portions from 40%, 20%, 10% OptiPrep™ solution and finally 2.8 mL of 5% OptiPrep™ solution in a polyallomer tube. Exosome pellet obtained from ultra-centrifugation was layered on top of 5% OptiPrep™ solution and centrifuged at 100,000 × g for 18 h at 4°C. After centrifugation 1 mL gradient fractions were collected from top to bottom and were diluted with 1.5 mL PBS and re-centrifuged at 100,000 × g for 1 h at 4°C. The resulting pellets were characterized by Western blotting and confocal microscopy.

### Electron microscopy

Blood was drawn from four healthy donors into K_3_EDTA collection tubes and plasma separated as described above. Exosomes were isolated from 1 mL of plasma as previously described and exosome pellet was suspended in 1 mL of PBS. Electron microscopic studies were carried out at the University of Nebraska Medical Center’s electron microscopy core facility using FEI Tecnai G2 Spirit transmission electron microscope operated at 80 kv. Isolated exosomes (10 μL) were placed on Silicon monoxide coated 200-mesh copper grids and negatively stained with NanoVan®, (Nanoprobes Inc., Yaphank, NY, USA).

### Confocal microscopy

Exosomes were isolated from 1 mL of plasma using Invitrogen Total Exosome isolation (from plasma) kit as described above. Isolated exosomes were suspended in 1 mL of PBS and treated with Quant-iT™ PicoGreen^®^ dsDNA reagent (3 μL/mL) at room temperature (22°C) for 1 hour. Fluorescence labeled exosomes (30 μL) were centrifuged at 1500 rpm for 10 min on glass slides using Shandon Cytospin® 3 cytocentrifuge. Slides were dried and coverslips were mounted onto slides with UltraCruz™ mounting medium (#sc-24941) and were analyzed by a Zeiss LSM 710 laser scanning confocal microscope (Oberkochen, Germany). In another experiment exosomes were isolated using density gradient centrifugation as described above and exosome pellet from each fraction was suspended in 100 μL of PBS and divided into two equal aliquots. One aliquot was treated with 5 μL of DNase enzyme (Promega RQ1 DNase cat # 9962606L) and 6 μL of buffer and the other aliquot with 11 μL of PBS and incubated at 22°C for 30 min. After this incubation 1.5 μL of10 times diluted Quant-iT™ PicoGreen^®^ dsDNA reagent was added to both aliquots and incubated at 22°C for 1 h. Labeled exosomes (61 μL) were centrifuged at 1500 rpm for 10 min on glass slides using Shandon Cytospin® 3 cytocentrifuge. Slides were dried and coverslips were mounted onto slides with UltraCruz™ mounting medium (#sc-24941) and were analyzed by Zeiss LSM 710 laser scanning confocal microscope.

### Quantification and sizing of exosomes

Exosome enumeration and sizing was carried out using the NanoSight NS300 instrument (Nanosight, UK) following the manufacturer’s protocol. The instrument uses a laser light source to illuminate nanoscale particles (10–1000 nm) which are seen as individual point-scatters moving under Brownian motion. The paths of the point scatters, or particles, are calculated over time to determine their velocity which can be used to calculate their size independent of density. The image analysis NTA software compiles this information and allows the user to automatically track the size distribution and number of the nanoparticles. The NanoSight NS300 instrument is also equipped with a 532 nm (green) laser diode to excite suitable fluorophores whose fluorescence can then be determined using a matched 565 nm long-pass filter. When measurements are made in fluorescence mode with a long-pass filter, only fluorescent emitting particles are measured. Real time tracking of individual fluorescently labeled particles helps determine size and concentration of labeled particles. Under light scatter mode, the total number of particles can be measured and subsequently compared to the concentration of labeled particles. Exosomes re-suspended in PBS were further diluted with PBS (5 times) in order to have the exosome concentration in the working range for the NanoSight NS300 (2 × 10^8^–8 × 10^8^) and then quantified and sized. Exo-FITC™ universal stain (SBI System Biosciences, Palo Alto, CA) has FITC fluorescent molecules conjugated to a protein known to universally bind exosomes. Exosomes were labeled with this exosome specific probe following manufacturer’s protocol and enumeration and sizing carried out using NanoSight NS300 instrument. Plasma exosomes were also stained with Quant-iT™ PicoGreen^®^ dsDNA reagent. Exosomes suspended in PBS were incubated with Quant-iT™ PicoGreen^®^ dsDNA reagent (3 μL/mL) for 1 hour at room temperature and analyzed by NanoSight NS300 instrument.

### Western blot analysis

Exosomes were isolated from human blood plasma (0.5 mL) as described above and suspended in cold protein lysis buffer (50 mM Tris pH 8.0, 140 mM NaCl, 1.5 mM MgCl2, 0.5% NP-40, 0.1% BSA, 10ug/ml aprotinin, 10ug/ml leupeptin, 1ug/ml pepstatin, 1mM PMSF). Protein samples were denatured and loaded onto 4–12% SDS-PAGE, electrophoresed and transferred to PVDF membranes. Membrane was blocked using 3% BSA in TBST at room temperature for 1 hour. The membranes were then incubated with either anti-CD63 antibody (rabbit anti-human, cat. # EXOAB-CD63A-1), anti-CD9 antibody (rabbit anti-human, cat. # EXOAB-CD9A-1),anti-Hsp70 antibody (rabbit anti-human, cat. # EXOAB-Hsp70A-1) (System Biosciences SBI, Palo Alto, CA, USA), anti-CD235a antibody (Glycophorin A antibody, cat. # GTX100300; GeneTex Inc. USA), anti-CD41 antibody (CD41 antibody[N2C1] internal, cat # GTX113758; GeneTex Inc. USA) or anti-CD45 antibody (CD45 antibody [GT0014], cat. # GTX628507; GeneTex Inc. USA) at 4°C overnight: Antibodies were diluted as per manufacturer’s recommendations; anti-CD63 antibody 1:1000, anti-HSP70 antibody 1:1000, anti-CD9 antibody 1:1000. Antibody binding was detected using HRP-conjugated anti-rabbit secondary antibody, followed by chemiluminescence detection (Bio-Rad, Hercules, CA, USA). Signals were visualized using LI-COR Odyssey-Fc Imaging System (LI-COR Biotechnology, Lincoln, NE).

### Extraction of DNA from cell-free plasma and exosomes

The QIAamp® Circulating Nucleic Acid Kit (Qiagen, Santa Clarita, CA) was used for the isolation of DNA from plasma, plasma exosomes and plasma supernatant obtained after exosome precipitation. The manufacturer’s recommended protocol was followed to isolate DNA from cell-free plasma (0.5 mL), plasma exosomes (isolated from 0.5 mL of plasma and re-suspended in 0.5 mL of PBS) and plasma supernatant obtained after exosome precipitation (0.9 mL). DNA extraction process included blanks where PBS was used instead of a sample. DNA was eluted in 50 μL of elution buffer and stored at -80°C until use. The values obtained for blanks were deducted from values obtained for test samples.

### Agarose gel separation of exosome DNA

Blood samples were pooled, plasma separated and exosomes isolated (from 7 mL of plasma) as described above. Exosome DNA was extracted as described in this manuscript. Eluted DNA was divided into two aliquots, one was treated with RNase enzyme and the other aliquot was not treated. Treated and untreated DNA was separated using 1% agarose gel, stained with GelRed nucleic acid stain (Biotium Inc.), and visualized using Bio-Rad ChemiDoc™ XRS+ system in two separate experiments. Relative amounts of exosome RNA and DNA in agarose gel were determined using ImageJ, a public domain Java-based image processing program.

### Analysis of exosome DNA by Agilent Bioanalyzer

Exosome DNA was extracted from blood as described above. DNA extracted from exosomes were treated with RNase as described above and analyzed using Agilent Bioanalyzer 2100 instrument and Agilent High Sensitivity DNA Kit following manufacturer’s recommended protocol. The Agilent 2100 Expert software analyze DNA profile of each sample automatically and displays electropherogram for each sample.

### Total DNA quantification

Qubit™ ds DNA HS assay kit and Qubit™ 3.0 fluorometer was used to quantify DNA isolated from cell-free plasma and plasma exosomes following manufacturer’s recommended protocol. Qubit™ RNA HS assay kit and Qubit™ 3.0 fluorometer was used to quantify RNA isolated from exosomes.

### Quantitative droplet digital PCR (ddPCR)

For plasma and plasma exosome DNA quantification, a ddPCR assay was designed which amplifies a short segment (76 bp) of human β-actin gene. Forward primer 5′-GCC AGG GCT TAC CTGG TAC ACT-3′ and reverse primer 5′-GTC ACA CTT GGC CTC ATT TTT-3′ were designed using Roche ProbeFinder online software. Probe for this assay is Roche’s universal probe library probe number 54 (cat. no. 04688511001) which was recommended by the ProbeFinder. Primers were purchased from Integrated DNA Technologies (IDT) (Coralville, IA). Universal probe number 54 was purchased from Roche. A PCR master mix, 2× ddPCR™ Supermix for Probes, was purchased from Bio-Rad Laboratories (Hercules, CA). Final concentrations of primers and probe in PCR reactions were 900 nM and 250 nM, respectively, in a final volume of 20 μL. The DNA template input volume was 5 μL. A Bio-Rad QX200 Droplet Digital™ PCR System was used as described by Hindson and colleagues [8]. Thermal cycling was performed with a Bio-Rad C1000 Touch Thermal cycler. The following PCR conditions were used: 95°C for 10 min, 40 cycles of 30 s at 94°C and 50 s at 60°C followed by a heating step at 98°C for 10 min to inactivate the polymerase. Data analysis was done using Bio-Rad QuantaSoft software version 1.7.4.0917.

### Comparison of DNA concentrations in cell-free plasma and plasma exosomes

Plasma was separated from each blood sample drawn into a 10 mL K_3_EDTA tube using the method described above. Cell-free plasma was aliquoted (0.5 mL) and divided into two groups. Group one was used to isolate DNA directly from plasma aliquots and group two was used to isolate DNA from exosomes isolated from plasma aliquots. DNA was extracted from plasma and exosomes as described above. DNA was quantified by Qubit™ ds DNA HS assay and ddPCR assay as described above.

### Effect of blood storage on plasma exosome and exosome DNA concentrations

Blood was drawn into 10 mL K_3_EDTA tubes and aliquoted to 4, 2.5 mL aliquots and stored at 22°C. Plasma separated from each aliquot at days 0, 3, 7 and 14, then exosomes isolated and a portion was used to enumerate the exosome number using the NanoSight instrument. The other portion was used to extract DNA and quantify using ddPCR.

### Statistical analysis

Statistical analysis was carried out using GraphPad Quick Calcs t test calculator online software (http://www.graphpad.com/quickcalcs/ttest1.cfm). Analysis was performed using paired, two-tailed Student's t-test and p < 0.05 was considered statistically significant.

## Results

### Characterization of human plasma exosomes by Western blotting and electron microscopic image analysis

**Fig 1:**
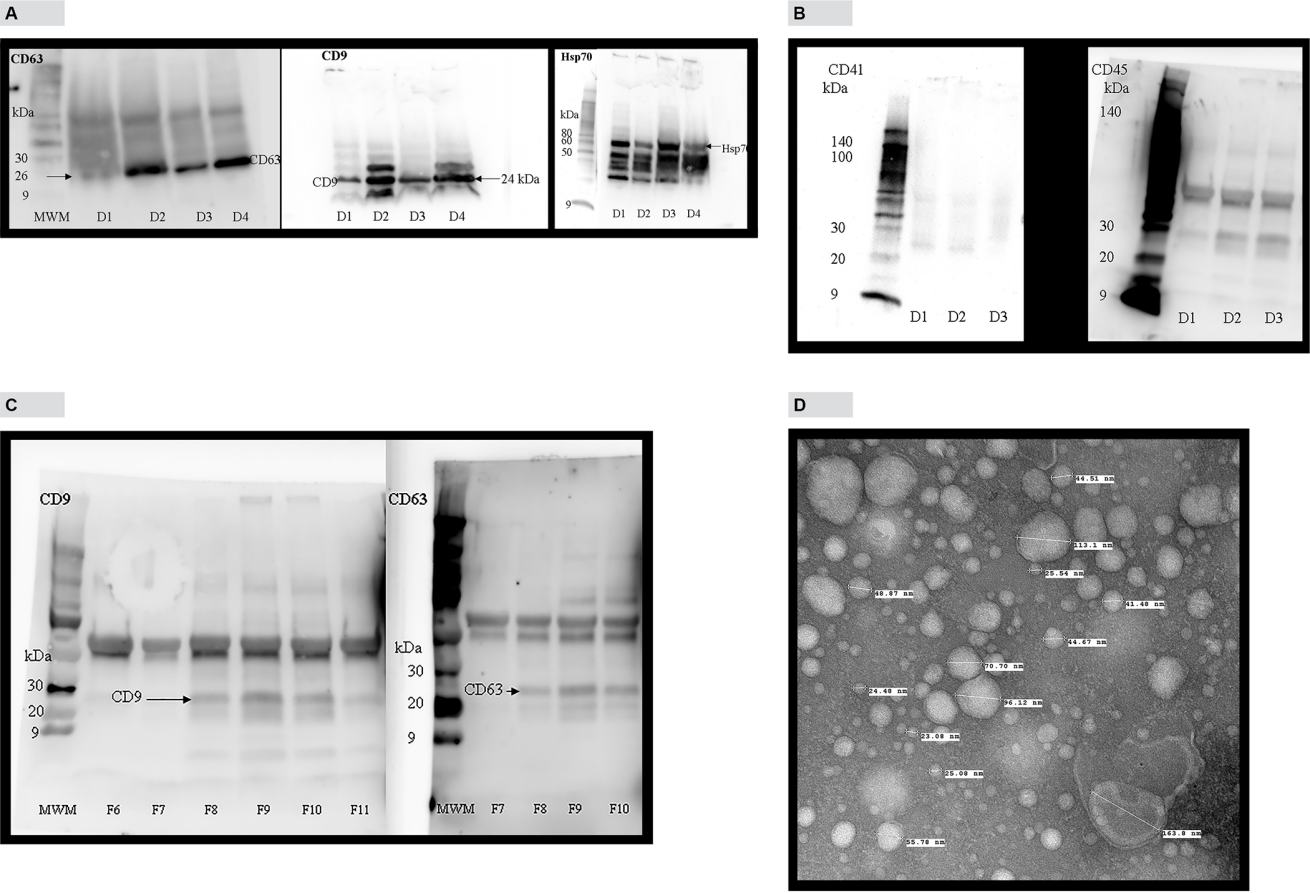
Characterization of plasma exosomes by Western blotting and transmission electron microscopy. A, Western blot analysis of exosome marker proteins, Hsp70 (MW 70 kDa), CD63 (MW 26 kDa) and CD9 (MW 24 kDa) in 4 donors. B, Western blot analysis of exosomes for microvesicles marker proteins, CD41 (MW 113 kDa) and CD45 (MW 147 kDa) in 3 donors. C, Analysis of density gradient fractions for CD9 and CD63 proteins. D, Electron microscopic analysis of exosomes isolated from human blood plasma, contrasted and embedded as described in Methods and Materials section. Note their cup shape, appearance, and heterogeneous size ranging from 15–165 nm. Magnification110000 ×.

Extracellular vesicles isolated from human blood plasma using Invitrogen Total Exosome isolation (from plasma) kit were analyzed for the presence of exosome marker proteins, Hsp70, CD9 and CD63. “Fig 1A” shows the presence of CD9, CD63 and Hsp 70 proteins in exosomes isolated from the plasma of four healthy donors. We also analyzed the exosomes isolated by the above method for the presence of CD41 and CD45 antigens which are reported to be concentrated in microvesicles [9]. According to “Fig 1B, detectable amounts of CD41 and CD45 antigens were not found in exosome pellet. “Fig 1C” is a Western blot analysis of fractions obtained from density gradient method showing the enrichment of exosome marker proteins CD9 and CD63 in fractions 8–10. We used transmission electron microscopy to determine size distribution, shape and morphology of exosomes isolated from human blood plasma using precipitation method. For the four samples analyzed, exosome sizes ranged from 17–170 nm. “Fig 1D” is a representative image which shows intact vesicles with classic exosome morphology and cup shape. This size distribution is generally consistent with published results for exosomes.

### Detection of dsDNA in plasma exosomes using confocal microscopy

**Fig 2:**
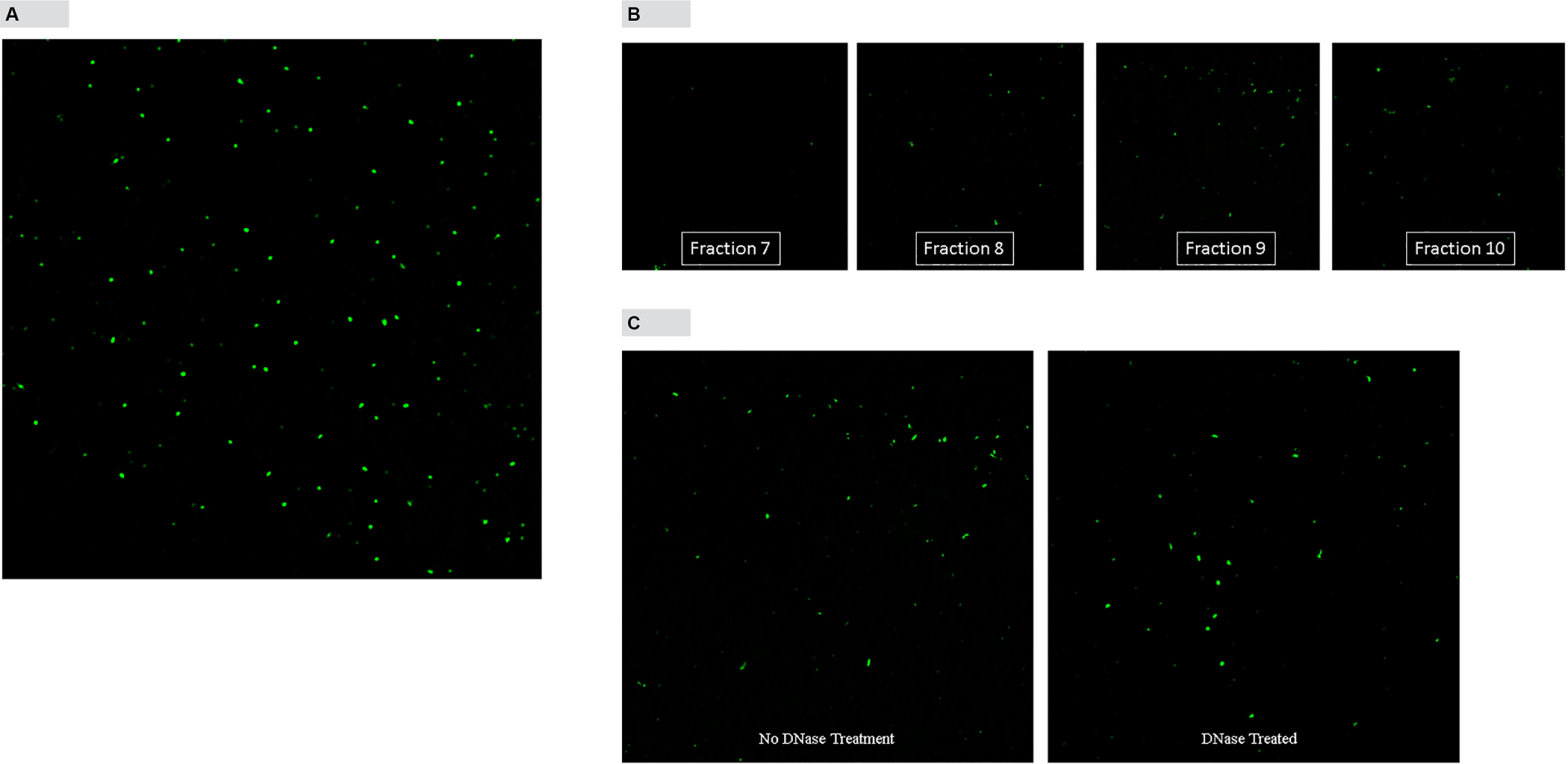
Fluorescence images of exosomes stained with Quant-iT™ PicoGreen^®^ dsDNA reagent under confocal microscopy. A, Bright green fluorescence of exosomes isolated using Invitrogen kit indicate the presence of dsDNA. B, Confocal image analysis of exosomes in fractions obtained by density gradient centrifugation. C, Confocal image analysis of exosomes with or without DNase treatment. Magnification 64 ×.

Exosomes isolated using Invitrogen Total Exosome isolation (from plasma) method were stained with Quant-iT™ PicoGreen^®^ fluorescence dye and examined under confocal microscope. “Fig 2A” shows confocal microscopic image of exosomes with intense green fluorescence indicating the presence of dsDNA in exosomes. Exosomes isolated by density gradient centrifugation method were also analyzed by confocal microscopy. Exosome pellets obtained from density gradient fractions were stained with Quant-iT™ PicoGreen^®^ fluorescence dye and analyzed by confocal microscopy. Only fractions 8, 9 and 10 which showed CD9 and CD63 enrichment, showed the presence of dsDNA (“Fig 2B”). In another experiment exosomes isolated from fraction 9 were either treated or not treated with DNase before staining with Quant-iT™ PicoGreen^®^ fluorescence dye and analyzing by confocal microscopy. Confocal results show that even after the DNase treatment dsDNA remained inside exosomes, indicating that dsDNA is present inside the exosomes (”Fig 2C”).

### Quantification and sizing of exosomes using NanoSight particle counter

**Fig 3:**
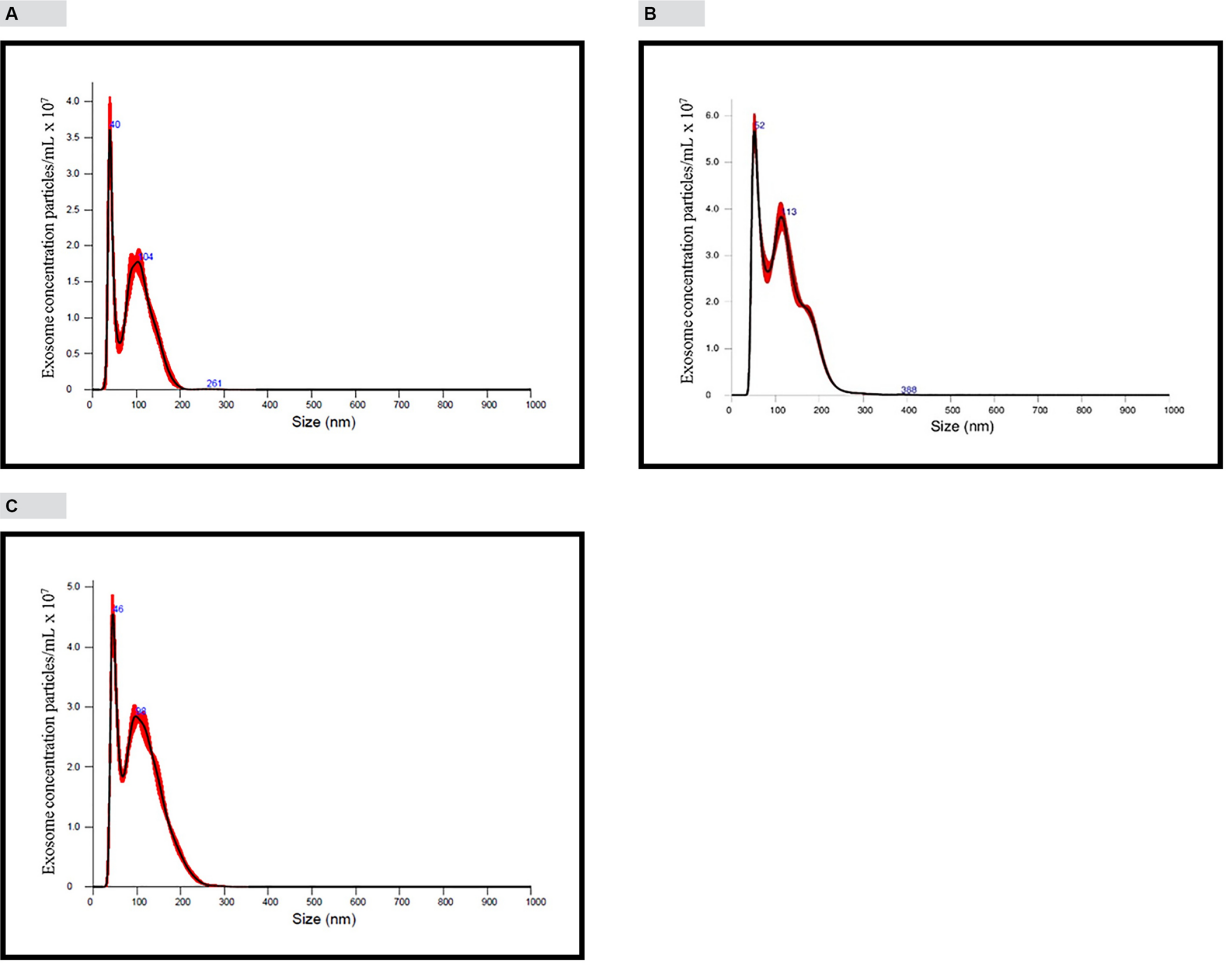
Quantification and sizing of exosomes using NanoSight NS300 particle counter and analysis of protein exosome markers by western blotting. For “Fig 3”, panels A, B and C, x-axis represents particle size and y-axis represents the mode peak particle concentration. Mode peak concentration value is less than the total particle concentration. The total particle concentration is represented by the areas under the curves. Software used in the instrument automatically calculate the areas under the curves and gives estimated total particle concentration. Since sample was diluted 5-times, concentration value provided by software was multiplied by five. A, Exosomes were analyzed under light scatter mode. Size is heterogeneous ranging from 30–260 nm. Mean size is 92.6 nm and mode is 39.7 nm. Concentration 9.25 × 10^9^ particles/mL. B, Analysis of Exo-FTIC™ (green fluorescence) labeled exosomes using fluorescence mode. Exosomes are heterogeneous in size ranging from 30–260 nm. Mean size is 113.3 nm and mode is 51.6 nm. Concentration 24 × 10^9^ particles/mL plasma. C, Analysis of exosomes stained with Quant-iT™ PicoGreen^®^ dsDNA reagent using fluorescence mode. Exosomes are heterogeneous in size ranging from 30–260 nm. Mean size is 106.5 nm and mode is 45.5 nm. Concentration 18 × 10^9^ particles/mL plasma.

“Fig 3A” shows exosomes analyzed on the NanoSight NS300 instrument under light scatter mode. NanoSight results show a size distribution ranging from 30–260 nm (mean 92.6 nm and mode 39.7 nm) and a concentration of 9.25 × 10^9^ vesicles/mL of plasma. “Fig 3B” shows the analysis of plasma exosomes stained with Exo-FITC™ universal stain. It shows two prominent peaks at 52 nm and 113 nm (mean 113.3 nm and mode 51.6 nm) and a concentration of 24 × 10^9^ vesicles/mL of plasma. Plasma exosomes stained with dsDNA staining dye Quant-iT™ PicoGreen^®^ showed a size distribution 30–260 nm (mean 106.5 nm and mode 45.5 nm) and a concentration of 18 × 10^9^ vesicles/mL of plasma (“Fig 3C”).

### Analysis of exosome DNA by agarose gel electrophoresis

**Fig 4:**
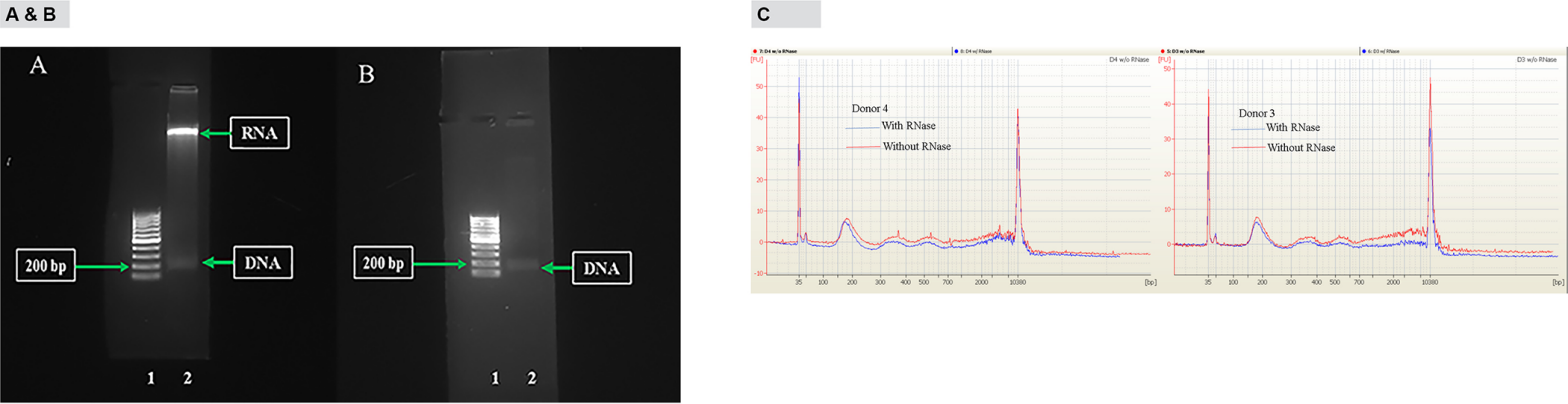
Analysis of exosome DNA by agarose gel separation and Agilent Bioanalyzer. A, Exosome DNA without RNase treatment. B, Exosome DNA with RNase treatment. High molecular weight band is removed by RNase treatment indicating that band represents RNA. Low molecular weight band is resistant to RNase treatment indicating that it is DNA. Majority of exosome DNA are in 200 bp size range. C, Overlaid Agilent 2100 Bioanalyzer electropherograms. Exosome DNA was extracted from two individual donors. Exosome DNA from both donors were either treated with RNase or not treated. RNase treated and not treated DNA were analyzed by Agilent Bioanalyzer and RNase treated and not treated electropherograms were overlaid.

“Fig 4A” shows exosome DNA without RNase treatment. According to “Fig 4A”, there are two clearly visible bands, one high intensity high molecular weight band and a moderate intensity low molecular weight band (~ 200 bp). Analysis of the agarose gel in “Fig 4A” by Densitometry, showed that the relative amount of exosome RNA is 5-fold higher than exosome DNA amount. Quantification of DNA and RNA extracted from exosome pellet using fluorescence based assays showed that RNA concentration in exosomes are 20–30-fold higher compared to exosome DNA concentration (data not shown). In addition to these two prominent bands, there seems to be other bands in between two prominent bands which are very hard to visualize. “Fig 4B”, shows exosome DNA treated with RNase enzyme. It is clear that RNase treatment removed a significant amount of high intensity high molecular weight band indicating that band is RNA co-purified during DNA extraction. RNase treatment had no visible effect on low intensity low molecular weight band indicating that band is DNA present in exosomes. Careful examination of “Fig 4B” shows other less visible DNA bands in addition to ~ 200 bp band. RNase treated or not treated exosome DNA was also analyzed by Agilent Bioanalyzer using Agilent High Sensitivity DNA kit. Fig 4C shows results from two donors. Electropherograms for with and without RNase were overlaid. The main exosome DNA peaks in donor 3 and donor 4 were 178 and 160 bp long fragments, respectively. In addition to this main peak there were three other minor peaks, around 350, 550 and 6000 bp range. Fig 4C shows RNase treatment had very little effect on exosome DNA profile.

### Comparison of total DNA concentration in plasma and plasma exosomes

**Fig 5:**
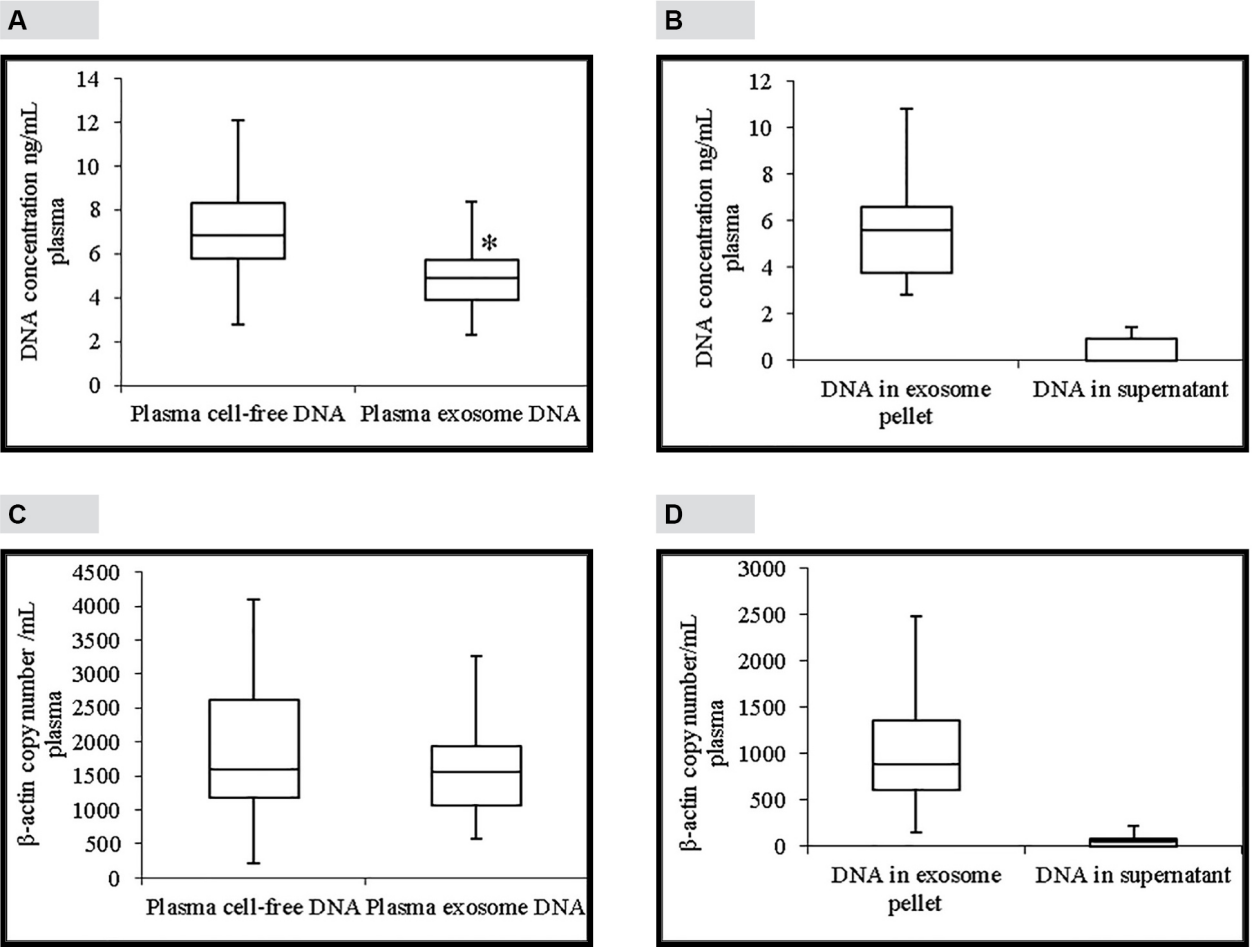
Total and amplifiable DNA concentrations in plasma and plasma exosomes. A, Comparison of total DNA concentrations in plasma (median 6.86 ng/mL) and plasma exosomes (median 4.9 ng/mL). B, Comparison of total DNA concentrations in exosome pellet (median 5.6 ng/mL) and plasma supernatant (median 0.0 ng/mL). C, Comparison of β-actin DNA concentrations in plasma and plasma exosomes detected by a ddPCR assay. There was no statistically significant difference between β-actin DNA concentrations in plasma (median 1600 β-actin copies/mL plasma) and plasma exosomes (median 1560 β-actin copies/mL plasma). D, Comparison of β-actin DNA concentrations in plasma exosome pellet (median 888 β-actin copies/mL plasma) and plasma supernatant (median 52 β-actin copies/mL plasma) detected by ddPCR assay. The line inside of the box indicates median value. The limits of the box represent the 75th and 25th percentiles. The whiskers indicate the 10th and 90th percentiles. Panels A and C; n = 23. Panels B and D; n = 16. * *p* < 0.01.

“Fig 5A”, shows a comparison between total DNA concentrations in plasma and plasma exosomes. Total DNA concentration in plasma ranges from 2.78–14.2 ng/mL plasma (median 6.86 ng/mL plasma; mean 7 ng/mL plasma). According to “Fig 5A”, total DNA concentration in plasma exosomes ranges from 2.3–12.3 ng/mL plasma (median 4.9 ng/mL plasma; mean 5 ng/mL plasma). This shows that plasma DNA concentration is a little higher than exosome DNA concentration and that difference is statistically significant (p = 0.01). “Fig 5B” compares total DNA concentrations in exosome pellet and supernatant. Total DNA concentrations in exosome pellets range from 2.8–17.8 ng/mL plasma (median 5.6 ng/mL plasma; mean 6 ng/mL plasma). Total DNA concentrations in plasma supernatants range from 0.0–3.4 ng/mL plasma (median 0.00 ng/mL plasma; mean 0.6 ng/mL plasma). This shows that about 90% of total DNA in plasma is localized in plasma exosomes.

### Comparison of amplifiable DNA concentration in plasma and plasma exosomes

Amplifiable DNA concentrations in plasma and plasma exosomes was determined by amplifying a 76 bp fragment of β-actin gene using a ddPCR assay. This assay was optimized for annealing temperature, primer concentration and probe concentration (data not shown). Linearity of this assay was determined by R^2^ value which was 0.9934. “Fig 5C” shows a comparison between amplifiable DNA concentrations in plasma and plasma exosomes. Amplifiable DNA concentration in plasma ranges from 220–4100 β-actin copies/mL plasma (median 1600 β-actin copies/mL plasma; mean 1896 β-actin copies/mL plasma). Amplifiable DNA concentration in plasma exosomes ranges from 575–4040 β-actin copies/mL plasma (median 1560 β-actin copies/mL plasma; mean 1660 β-actin copies/mL plasma). There was no statistically significant difference between amplifiable DNA concentrations in plasma and plasma exosomes. “Fig 5D”, shows a comparison of amplifiable DNA concentrations in exosome pellet and plasma supernatant. Amplifiable DNA concentration in plasma exosome pellet ranges from 156–2480 β-actin copies/mL plasma (median 888 β-actin copies/mL plasma; mean 991 copies/mL plasma). Amplifiable DNA concentrations in plasma supernatant range from 0.0–280 β-actin copies /mL plasma (median 52 copies/mL plasma; mean 74 copies/ml plasma). This shows that about 93% of amplifiable DNA in plasma is localized in plasma exosomes.

### Effect of blood storage on exosome and exosome DNA concentrations

**Fig 6:**
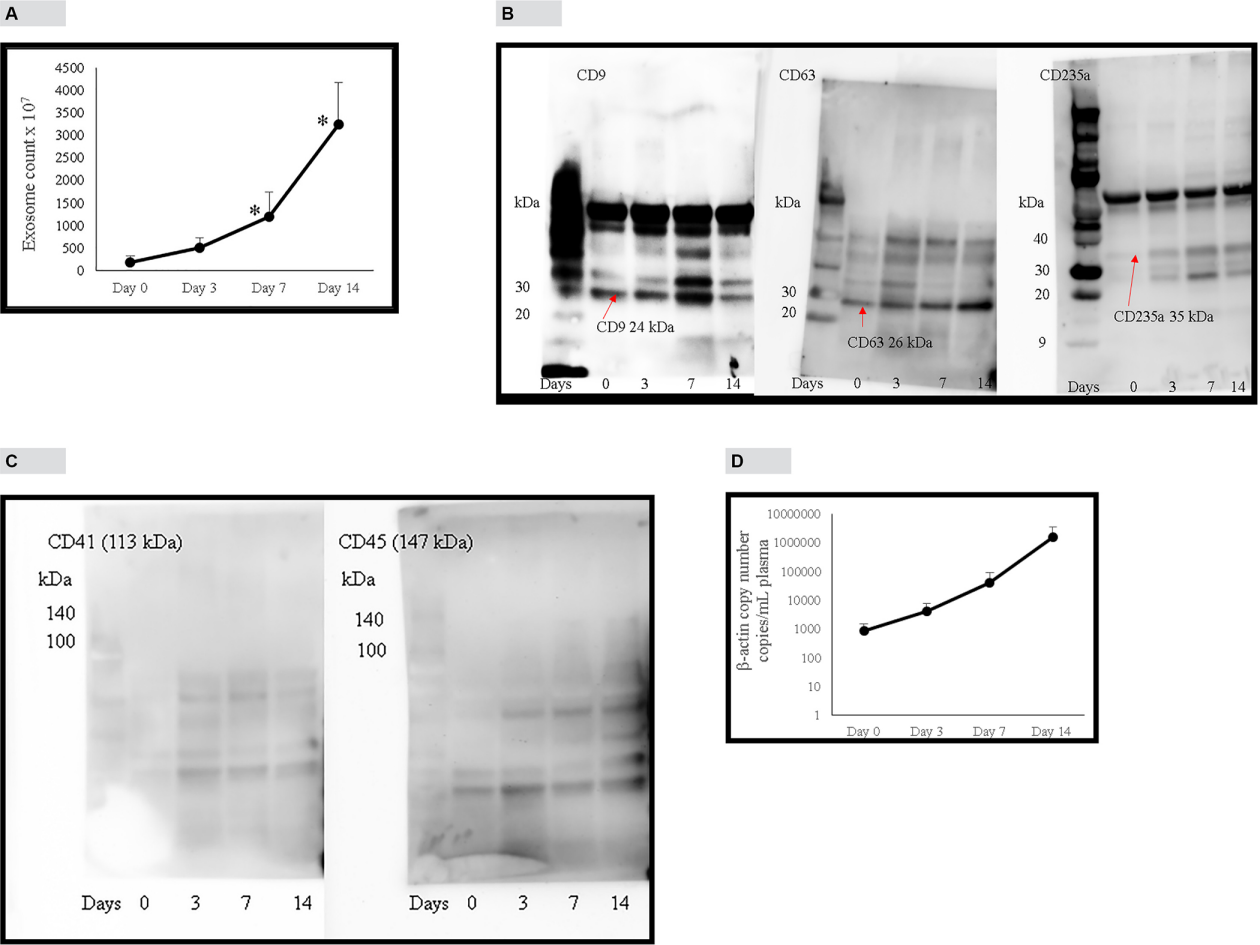
Effect of blood sample storage (at 22°C) on exosome and exosome DNA concentration in plasma. A, Effect of blood storage on exosome concentration analyzed by NanoSight instrument under light scatter mode. Blood from each donor was divided into 4 aliquots and stored at 22°C. Plasma separated from each aliquot at indicated days, exosomes isolated and enumerated. B, Effect of blood storage on proteins CD9, CD63 and CD235a in plasma exosome pellet (isolated by Invitrogen method). C, Effect of storage on proteins CD41 and CD45 in plasma exosome pellet. D, Effect of blood storage on exosome DNA concentration as detected by β-actin ddPCR assay. Blood from each donor was divided into 4 aliquots and stored at 22°C. Plasma separated from each aliquot at indicated days, exosomes isolated. DNA extracted from exosomes and β-actin copy number detected by ddPCR assay. Error bars indicate SD. Panel B is in logarithmic scale.

There was a steady increase in exosome concentration (exosomes were isolated using Invitrogen kit) in blood plasma upon storage. According to “Fig 6A”, initial exosome concentration was 184 × 10^7^ particles/mL plasma. At days 3, 7 and 14 there were 3, 7 and 18-fold increases in exosome concentrations, respectively. Western blotting was used to analyze CD9, CD63 and CD235a (an antigen specific for red blood cells) proteins in exosomes isolated from stored blood samples. “Fig 6B” shows that initial concentrations of proteins CD9 and CD63 increase over time while red blood cell specific protein, CD235a shows undetectable concentrations at the beginning yet increase over time. This shows that when blood was drawn, there was very low concentrations of red cell-derived exosomes in blood but increase when blood is stored for longer periods of time. CD41 and CD45 proteins which originate from platelets and leucocytes, respectively are mainly present in microvesicles [9]. “Fig 6C” shows that CD41 and CD45 proteins are absent in exosomes isolated from stored blood samples indicating that exosomes isolated using Invitrogen kit was not contaminated with microvesicles. Exosome DNA concentration also increased upon storage concomitant with exosome concentration increase (“Fig 6D”). Initial β-actin DNA concentration was 903 copies/mL plasma. At days 3, 7 and 14 there were 5, 46 and 1753-fold increase in β-actin copy number, respectively.

## Discussion

According to previously published reports, size of exosomes range from 40–100 nm and has a specific morphology and shape [9, 10]. Plasma exosomes isolated using Invitrogen exosome isolation kit showed shape and morphology under electron microscope that are characteristic of exosomes (Fig 1D). Analysis of exosomes isolated using Invitrogen kit by NanoSight instrument showed the mean size of 92.6 nm and mode of 39.7 nm which is generally consistent with published results for plasma exosomes [11]. Exo-FITC™ stain is a conjugate of a protein which specifically binds with exosome membrane proteins and FITC fluorescence dye. Analysis of exosomes labeled with Exo-FITC™ stain by NanoSight confirms that particles isolated from plasma are exosomes. Sizing of plasma exosomes using NanoSight light scatter mode showed a mean of 92.6 nm and a mode of 39.8 nm. Sizing of the same sample stained with Exo-FITC™ under fluorescence mode showed a mean of 113.3 nm and mode of 51.6 nm. These results confirm that vesicles isolated from plasma are exosomes. When plasma exosomes isolated by Invitrogen kit were analyzed by Western blotting, it showed the presence of cytocolic protein Hsp 70, commonly present in exosomes and membrane-bound tetraspanins, CD9 and CD63 that are known to be enriched in exosomes [7, 9, 12]. Membrane-bound tetraspanins, CD41 and CD45 which are enriched in microvesicles [9] could not be detected in exosomes isolated by the kit (Fig 1B) indicating that exosomes isolated were relatively free of microvesicles. Exosomes were also isolated using a previously described density gradient centrifugation method. Exosome enriched fractions were identified using Western blotting analysis of tetraspanins, CD9 and CD63 that are enriched in exosomes Exosome markers CD9 and and CD63 were enriched in fractions 8–10 corresponding to a buoyant density of 1.16 to 1.39 (Fig 1C). These results are comparable to previously published results for exosomes [7, 13].Ashley et al. [14] have reported that they have used Quant-iT™ PicoGreen^®^ fluorescence dye to detect mitochondrial DNA depletion in living human cells. According to their experiments, Quant-iT™ PicoGreen^®^ fluorescence dye can penetrate cell and mitochondrial membranes and stain dsDNA at a concentration of 3 μL/mL at 37°C within 1 hour. We used their protocol to stain exosome DNA with Quant-iT™ PicoGreen^®^ dye. Analysis of plasma exosomes stained with Quant-iT™ PicoGreen^®^ fluorescence dye by NanoSight fluorescence mode and confocal microscopy shows the presence of dsDNA in exosomes. This is further confirmed by agarose gel electrophoresis of DNA isolated from plasma exosomes. Separation of DNA isolated from plasma exosomes showed two prominent bands, one high intensity and high molecular weight and the other a low molecular weight band. However, treatment of the DNA sample with RNase removed most of the high molecular weight band indicating that it is RNA co-purified during exosome DNA extraction. This is further confirmed by Bioanalyzer results (Fig 4C) which shows no high molecular weight high intensity band because the Bioanalyzer kit we used detects only DNA not RNA. Densitometric analysis of the agarose gel in Fig 4A shows that the relative amount of exosome RNA is 5-fold higher than the amount of exosome DNA. However, extracting DNA and RNA from exosomes and quantifying using fluorescence probes specific for DNA and RNA showed RNA concentrations in exosomes are 20–30-fold higher compared to exosome DNA concentration (data not shown). The low molecular weight band (~200 bp in length) which was resistant to RNase digestion is the main DNA band in exosomes. This is further confirmed by Bioanalyzer results which shows that major exosome DNA peak is less than 200 bp long and high molecular weight DNA is minimal (Fig 4C). This observation is consistent with the results published by other investigators. In a 2010 study, Fan and colleagues used paired-end sequencing of all the cell-free DNA in plasma to determine the size distribution and found the majority of cfDNA fragments were ~ 162 bp in length [15]. An editorial written by Hahn and Zimmermann summarizes recent work related to cfDNA fragment size distribution [16]. A recent comprehensive review article by Thierry et al. reviews the origin, structures, and functions of cfDNA [17]. In this review, authors discuss the size distribution of cfDNA obtained from cancer patients and shows that cfDNA from cancer patients is highly fragmented compared to cfDNA from healthy individuals. They have shown that cfDNA from cancer patients is < 145 bp in length. Using this observation, they call into question apoptosis as the only origin of cfDNA. A recent study conducted by Yu et al. using paired-end sequencing has shown that size-based molecular technologies could be used to detect chromosomal aneuploidy [18]. A Similar study published in 2016 also shows utilization of cfDNA fragment size distribution in predicting prenatal copy number variation [19]. A review article summarizes the technologies available for cfDNA fragment size analysis and utility of size profiling in clinical test development [20]. A comparison of plasma cfDNA and plasma exosome DNA concentrations using a fluorescence dye method and a ddPCR method gave conflicting results. According to fluorescence dye method, plasma cfDNA concentration was slightly higher than plasma exosome DNA concentration which showed a statistically significant difference. However, detection of amplifiable plasma cfDNA and plasma exosome DNA concentrations using ddPCR showed no such difference. Since plasma is a very complex mixture, extraction of cfDNA directly from plasma may give a DNA sample contaminated with some impurities. We suggest that slightly higher plasma cfDNA concentration results from the interaction of these impurities with the fluorescence dye or there may be highly fragmented, very short dsDNA fragments outside the exosome. DNA concentration studies in exosome pellet and plasma supernatant show that 90% of total cfDNA and 93% of amplifiable DNA in plasma are located in exosomes. In this study, we have demonstrated that plasma cfDNA is located in membrane bound exosomes protecting DNA from nuclease mediated degradation. This explains the observation of Norton et al. [21] that cfDNA concentration is stable in cell-free plasma for up to 14 days at room temperature. We also demonstrated that post phlebotomy storage of blood samples increased plasma exosome and exosome DNA concentrations over time indicating release of exosomes by blood cells during storage. Western blot analysis of exosomes isolated from stored blood samples shows that concentrations of tetraspnins CD9 and CD63 which are enriched in exosomes increase over time (Fig 6B). However, tetraspanins that are enriched in microvesicles, CD41 and CD45 could not be detected in exosomes isolated from stored blood samples (Fig 6C) indicating that exosome preparations are relatively free of microvesicles. Tetraspanin CD243a is a red cell membrane protein and Western blot analysis shows that CD243a concentration is very low in day 0 blood samples (Fig 6B).However over time CD243a concentration increase indicating red cells release exosomes during storage. It would be interesting to investigate whether exosomes released from red cells contain DNA or not. This study provides new information that a large proportion of plasma cfDNA is located in exosomes. This new information may be useful for the development of new noninvasive screening, diagnostic, and prognostic tests using fetal and tumor cfDNA found in maternal and cancer patient’s blood, respectively.
